# The *Atg16l1* gene: characterization of wild type, knock-in, and knock-out phenotypes in rats

**DOI:** 10.1152/physiolgenomics.00114.2020

**Published:** 2021-04-05

**Authors:** Kari L. Chesney, Hongsheng Men, Miriam A. Hankins, Elizabeth C. Bryda

**Affiliations:** ^1^Comparative Medicine Program, College of Veterinary Medicine, University of Missouri, Columbia, Missouri; ^2^Department of Veterinary Pathobiology, University of Missouri, Columbia, Missouri; ^3^Rat Resource and Research Center, University of Missouri, Columbia, Missouri; ^4^Animal Modeling Core, University of Missouri, Columbia, Missouri

**Keywords:** CRISPR, Crohn's disease, genetic models, inflammatory bowel disease, phenotyping

## Abstract

*ATG16L1* is a ubiquitous autophagy gene responsible, in part, for formation of the double-membrane bound autophagosome that delivers unwanted cellular debris and intracellular pathogens to the lysosome for degradation. A single, nonsynonymous adenine to guanine polymorphism resulting in a threonine to alanine amino acid substitution (T300A) directly preceded by a caspase cleavage site (DxxD) causes an increased susceptibility to Crohn’s disease (CD) in humans. The mechanism behind this increased susceptibility is still being elucidated, however, the amino acid change caused by this point mutation results in increased ATG16L1 protein sensitivity to caspase 3-mediated cleavage. To generate novel rat strains carrying genetic alterations in the rat *Atg16l1* gene, we first characterized the wild-type rat gene. We identified four alternative splice variants with tissue-specific expression. Using CRISPR-Cas9 genome editing technology, we developed a knock-in rat model for the human *ATG16L1* T300A CD risk polymorphism, as well as a knock-out rat model to evaluate the role of *Atg16l1* in autophagy as well as its potential effect on CD susceptibility. These are the first reported rat strains with alterations of the *Atg16l1* gene. Consistent with studies of the effects of human *ATG16L1* polymorphisms, models exhibit morphological abnormalities in both Paneth and goblet cells, but do not develop spontaneous intestinal permeability or inflammatory bowel disease. Analysis of the gut microbiota does not show inherent differences in bacterial composition between wild-type and genetically modified animals. These *Atg16l1* strains are valuable new animal models for the study of both autophagy and CD susceptibility.

## INTRODUCTION

Crohn’s disease (CD) is one of the two chronic inflammatory bowel diseases (IBD) that affect the lining of the gastrointestinal (GI) system. Familial linkage, twin studies, and previously established genome-wide association studies confirm the important role of genetics in IBD (1–4). More than 230 genetic alleles have been linked to CD, one of the most common of which lies in the autophagy-related 16-like 1 (*ATG16L1*) gene (5–8). Individuals who possess a single, nonsynonymous adenine to guanine polymorphism resulting in a threonine to alanine substitution directly preceded by a caspase cleavage motif in the ATG16L1 protein (ATG16L1 T300A) have an increased predisposition for CD over those who retain a threonine in this position (ATG16L1 T300) (9). This amino acid substitution causes increased sensitivity of the ATG16L1 protein to caspase 3-mediated cleavage during death-receptor activation and starvation-induced metabolic stress, resulting in decreased levels of full-length ATG16L1 protein and diminished macroautophagy, hereafter referred to as autophagy (10–12).

Autophagy is the evolutionarily conserved degradation pathway that delivers unwanted cellular debris to the lysosome for destruction (13–15). In the absence of stress, basal autophagy functions to eliminate and recycle damaged or long-lived cellular components that could otherwise become toxic. During periods of cellular stress, autophagy is critical in cell survival by encapsulating and degrading intracellular bacteria and damaged cellular products to maintain homeostasis. ATG16L1 is necessary for the formation of the double-membrane vesicle, the autophagosome, during autophagy (16–18). Without proper autophagosome formation, there is no autophagy.

The mechanism by which the *ATG16L1* T300A polymorphism causes increased susceptibility to CD is still incompletely understood. Previous studies examining the role of *Atg16l1* in autophagy and CD have found that mice hypomorphic for *Atg16l1* (Atg16l1^HM1^) and mice homozygous for the *ATG16L1* CD risk allele display the same abnormal Paneth cells granulation seen in patients with CD homozygous for the T300A polymorphism (10, 16). Furthermore, mice carrying the *Atg16l1* T300A risk allele exhibit increased retention of bacteria within polymorphonuclear cells, greater bacterial replication and dissemination throughout the gut, and *Salmonella*-induced colitis (19). To further the research on the association of *ATG16l1* and CD, our laboratory developed the first rat model carrying a CD risk allele, *Atg16l1* T300A (F344-*Atg16l1^em8Rrrc^*, referred to hereafter as T300A), as well as a knockout rat model of *Atg16l1* (SD-*Atg16l1^em2Rrrc^*, hereafter referred to as em2) using CRISPR-Cas 9 (43).

The following describes the genotypic and phenotypic characterization of wild-type (WT) rat *Atg16l1* as well as the characterization of rats heterozygous for the *Atg16l1* T300A risk allele and an *Atg16l1* knockout model. We identified four wild-type *Atg16l1* transcripts, two previously unknown, all with the capability to produce protein in vitro and with tissue-specific patterns of expression. We also show that our T300A rat strain has evidence of abnormal Paneth cell morphology and does not exhibit altered intestinal permeability, inherent gut microbiota differences compared with wild-type animals, nor spontaneous inflammatory bowel disease. Given these phenotypic similarities to human patients with CD, our T300A rat strain represents a useful new tool to further understand autophagy and its role in CD susceptibility in humans.

## MATERIALS AND METHODS

### Animals

All studies were performed in accordance with the *Guide for the Care and Use of Laboratory Animals* and were approved by the University of Missouri Institutional Animal Care and Use Committee. Rats were group-housed by sex and strain on the same ventilated rack with a maximum of four rats per 144 in.^2^ microisolator cage and at least one wild-type (WT) and one heterozygous (HET) rat per cage (Thoren Maxi-Miser Interchangeable System, Hazelton, PA). Environmental parameters included: 14:10-h light-dark cycle, humidity between 50%–70%, and temperature between 70°F–74°F. All animals were provided ad libitum autoclaved food (Lab Diet 5008, St. Louis, MO) and sulfuric acid treated water. Animals were confirmed to be free of adventitious viruses, parasites, and pathogenic enteric and respiratory bacteria through a quarterly dirty bedding sentinel monitoring program with diagnostic testing performed by IDEXX BioAnalytics (Columbia, MO).

### Creation of Rat Strains/Stocks

Both the em2 knockout and T300A knockin rat strains were developed using CRISPR-Cas9 genome editing technology by the Rat Resource and Research Center (Columbia, MO) and bred on site for these studies. Immature 4- to 5-wk-old Sprague-Dawley Hsd:SD (SD) and Fischer 344 F344/NHsd (F344) rats were purchased from Envigo (Indianapolis, IN). The rats were superovulated by intraperitoneal injection of 20 IU PG600 (Valley Vet Supply, Marysville, KS), followed by 40 IU human chorionic gonadotropin (hCG) (Calbiochem, San Diego, CA) 50 h later. Zygotes were collected 23–24 h after hCG from copulation plug positive females and then cultured in modified rat one cell embryo culture medium (mR1ECM) at 37°C, with 5% CO_2_, 5% O_2_, and maximal humidity (20). Two guide RNAs (gRNAs; Supplemental Table S1; see https://doi.org/10.6084/m9.figshare.12949244.v1), one targeting exon 5 and one targeting exon 13 of the rat *Atg16l1* gene, were designed. To generate animals carrying indels within the *Atg16l1* gene, pronuclear microinjection of both gRNAs (50 ng/µL each) and 100 ng/µL Cas9 mRNA into SD zygotes was performed. To generate the T300A knockin strain, a single-stranded oligonucleotide (ssODN) harboring a single base pair polymorphism of guanine to alanine in exon 10 of the rat *Atg16l1* gene, resulting in a threonine to alanine substitution at amino acid position 299 in the rat protein that mimics the human T300A variant was designed. This ssODN repair template (100 ng/µL) along with one gRNA (50 ng/µL) and Cas9 mRNA (100 ng/µL) were used for pronuclear microinjection into F344 zygotes. Following in vitro culture and no more than 1 h after injection, surviving zygotes were surgically transferred into pseudopregnant SD females. Resulting pups were screened at 2 wk of age for the genetic alterations of interest. DNA was extracted from tail-snips using the DNeasy Blood & Tissue Kit (QIAGEN, Valencia, CA). Indels were detected using the Surveyor assay (Integrated DNA Technologies, Coralville, IA) following the manufacturer’s protocol. High-resolution melt analysis with subsequent Sanger nucleotide sequencing were used to identify animals carrying the T300A polymorphism. Animals carrying the mutations of interest were bred to establish each strain/stock. The strains are available from the Rat Resource and Research Center (www.rrrc.us) as RRRC#896 (F344-*Atg16l1^em8Rrrc^*) and RRRC#897 (SD-*Atg16l1^em2Rrrc^*).

### RNA Transcript Analysis

#### Tissue samples.

Tissue samples were collected from six wild-type (WT) and six heterozygous (HET) rats from the em2 and T300A strains (3 males and 3 females per group) for RNA isolation. Tissues included: brain, thymus, esophagus, heart, lung, liver, kidney, spleen, stomach, jejunum, ileum, cecum, colon, and gonad. Animals were euthanized according to the 2013 *AVMA Guidelines for the Euthanasia of Animal* by CO_2_ asphyxiation and secondary cardiac exsanguination. Harvested tissues were immediately frozen in liquid nitrogen and stored at −80°C until processing.

#### RNA extraction.

Tissue from each organ (30 mg) was individually placed into a sterile Red RINO lysis tube (Next Advance, Troy, NY) containing 600 μL of Buffer RLT and 6 μL β-mercaptoethanol. Samples were mechanically disrupted using the Bullet Blender Storm Tissue Homogenizer (Next Advance) for 3 min at speed level 12. Samples were processed thereafter using the RNeasy Mini Kit (QIAGEN) following the RNeasy Kit protocol, including the optional on-column DNase digestion using DNaseI (Thermo Fischer Scientific, AM2222; Waltham, MA). RNA concentrations were determined using the Experion Automated Electrophoresis System and Experion RNA StdSens chips (Bio-Rad Laboratories, Hercules, CA). Only RNA samples with an RNA quality indicator (RQI) value of ≥8 (1–10 scale of quality) were used for transcript identification. RNA samples were stored at −80°C until further analysis.

#### Atg16l1 transcript identification.

Two-step RT-PCR was performed using the Superscript IV First-Strand Synthesis System (Thermo Fisher Scientific). Two (2) microgram of total RNA from each tissue sample and random hexamer primers (50 ng/μL) were used to produce cDNA according to the manufacturer’s protocol. Gene-specific amplification was achieved with FastStart Taq DNA Polymerase (Sigma, St. Louis, MO). Multiple gene-specific primer sets and amplification parameters were used to identify WT rat *Atg16l1* splice variants (Supplemental Table S2; see https://doi.org/10.6084/m9.figshare.12949238.v1). Amplified products were separated by gel electrophoresis on agarose gels in 1X Tris-acetate EDTA (TAE). Amplicons were gel-purified using the QIAquick Gel Extraction Kit (QIAGEN). DNA concentrations and purity were measured using the Nanodrop 8000 Spectrophotometer (Thermo Fisher Scientific) and samples were sent to the University of Missouri DNA Core (Columbia, MO) for nucleotide sequence analysis. Resulting sequences were analyzed with SnapGene software (GSL Biotech LLC, Chicago, IL) and compared with the reported full-length *Rattus norvegicus Atg16l1* sequence (Ensembl, *Rnor* v 6.0, ENSRNOT00000024445.3). Analysis to determine which alternative transcripts were present in both the em2 and T300A strains was performed using RNA from tissues from both WT and HET animals (*n* = 6 for each genotype for each strain/stock).

### Generation of Splice Variant Clones

cDNA representing each WT *Atg16l1* splice variant was cloned by In-Fusion cloning (Takara Bio Inc. Kusatsu, Shiga Prefecture, Japan) into the pEGFP-N1 vector (V12024; NovoPro Bioscience, Inc., Shanghai, China). The pEGFP vector was amplified using five sets of overlapping primers to verify for the proper sequence before use (Supplemental Table S4; see https://doi.org/10.6084/m9.figshare.12949235.v1). For our purposes, EGFP was removed from the plasmid during double-enzyme restriction digest and linearization using *EcoR*1-HF and *Not*I-HF restriction enzymes in NEB cutsmart buffer. Linearized vector was purified using the NucleoSpin Gel and PCR Cleanup Kit (Takara Bioscience Inc.).

Two sets of PCR primers (Supplementary Table S3; see https://doi.org/10.6084/m9.figshare.12949241.v1) were used to amplify each transcript in two fragments using CloneAmp HiFi PCR Premix (Takara Bio Inc.). Each fragment, or cloning insert, as well as the linearized vector, contained a 15 basepair overlap at their termini. This overlap allowed amplicons to be either joined to adjacent cloning inserts or to be joined to the linearized plasmid, ensuring proper orientation and placement within the plasmid. Amplicons were separated by gel electrophoresis and purified using the NucleoSpin Gel and PCR Clean-Up kit (Takara Bioscience Inc.). Nucleotide sequence analysis of each variant was performed by the University of Missouri DNA Core, and sequences were checked for accuracy using SnapGene software before cloning. An insert-to-vector cloning molar ratio of 2:2:1 (insert 1: insert 2: vector) was used to optimize cloning results. DNA cloning inserts, linearized vector, In-Fusion enzyme premix, and deionized water (10 μL total) were incubated at room temperature for 15 min and transformed into Stellar competent bacterial cells (Takara Bio Inc.). Transformed bacteria were grown on LB-agar containing 50 µg/mL kanamycin at 37°C. Plasmid DNA was isolated from individual bacterial transformants using a boiling lysis prep method (https://www.csun.edu/~mls42367/Protocols/B_lysis_plas_prep.pdf). Briefly, single colonies from LB-agar plates were incubated in LB media with kanamycin (50 μg/mL) overnight on a shaker plate at 37°C and 200 rpm. The following morning, bacterial cells were pelleted at 1,250 *g* at 4°C for 10 min and the supernatant was removed. Pellets were resuspended in 100 μL 1X STET buffer (10 mM Tris-HCl, 1 mM EDTA, 100 mM NaCl, 5% Triton X-100) containing 0.5 mg/mL lysozyme. Samples were incubated at 95°C for 30 s in a heat block. Samples were centrifuged at 14,000 *g* for 10 min at room temperature and pellets were discarded. Isopropanol (110 μL) was added to the supernatant and centrifuged at 14,000 *g* for 10 min at room temperature to collect the plasmid DNA. The supernatant was removed and the pellet was washed with ice cold 70% ethanol before resuspending in 20 μL TE buffer and stored at −20°C. Plasmid DNA was then screened by PCR for proper orientation of inserts. DNA from bacterial colonies with plasmids carrying the correct insertions were purified using the NucleoSpin Gel and PCR Clean Up Kit for transfection.

### Protein Expression

#### Cell transfection.

HEK293 cells (ATTC, Manassas, VA) were grown in 75 cm^2^ sterile flasks (Thermo Fisher Scientific) containing minimum essential media (Thermo Fisher Scientific), 10% fetal bovine serum (Sigma), 1% penicillin-streptomycin (Sigma) at 37°C, and 5% CO_2_. Cells were seeded into 25 cm^2^, sterile, vented cap, nontreated culture flasks (NEST Scientific; Rahway, NJ) at 700,000 cells/flask 24 h before transfection. Cells were between 70%–80% confluence at the time of transfection. Transfection was performed using the Lipofectamine 3000 reagent protocol (Invitrogen). Experimental groups included: each of the four splice variant overexpression plasmids (6.5 µg/flask), EGFP-N1 positive transfection control (6.5 µg/flask), lipofectamine reagent only (negative control) and cells only (negative control) to give a total of seven experimental groups performed in triplicate (24 flasks). Cells were incubated for 8 h at 37°C and 5% CO_2_ posttransfection and then the media were removed and replaced with 5 mL of fresh growth media to each flask. Cells were incubated for another 40 h.

#### Protein extraction.

After incubation, cells were collected by dissociation with 2 mL 1× TrypLE Express enzyme (Thermo Fisher Scientific) for 5 min at 37°C and placed into 8 mL of growth media in 15 mL conical tubes. Samples were subjected to centrifugation at 1,250 *g* for 10 min at 4°C. Cell pellets were washed with ice cold 1× PBS, spun again at 1,250 *g* for 10 min at 4°C and resuspended in 500 μL ice-cold RIPA lysis buffer [150 mM sodium chloride, 1.0% Triton X-100, 0.5% sodium deoxycholate, 0.1% sodium dodecyl sulfate (SDS), and 50 mM Tris-HCl pH 8.0] containing 0.1% fresh protease inhibitor cocktail (MFCD00677817; Sigma), 0.5 mM phenylmethylsulfonyl fluoride (36978, Thermo Fisher Scientific), and 20 mM sodium orthovanadate (MFCD00003511; Sigma). The samples were incubated on ice for 30 min with intermittent pipetting to fully lyse cells and release protein. Cell membranes were mechanically disrupted by pipetting the sample through a 1 mL pipette tip 10 times and vortexing for 10 s before incubation on ice for 30 min. Lysed samples were centrifuged at 14,000 *g* for 15 min at 4°C to pellet the cell debris, and the supernatants were transferred to microcentrifuge tubes and stored at −20°C.

Protein was measured using the Pierce BCA Protein Assay Kit (Thermo Fisher Scientific) using bovine serum albumin (BSA) standards in RIPA buffer. Briefly, reagent A and B were mixed in a 50:1 ratio with a resulting volume of 200 µL working reagent per sample. Working reagent and 10 µL of sample was added to each well (1:20 dilution of sample). The plate was covered and incubated at 37°C for 30 min. Absorbance was measured at 562 nm on the SpectraMax M3 microplate reader with SoftMax Pro 6.2 microplate data analysis software (Molecular Devices, Sunnyvale, CA).

#### Protein analysis.

Proteins were separated using ExpressPlus PAGE gels (10 × 8 cm, 12%, 12 well; GenScript, Jiangsu Province, China) at 140 V for 75 min following the manufacturer’s protocol. Gel electrophoresis was performed using 1× Tris-MOPS running buffer (M00138, Genscript), 1× transfer buffer (M00139, Genscript), and 5× sample buffer (MB01015, Genscript) against Precision Plus Protein Kaleidoscope Prestained Standards (Bio-Rad Laboratories).

Proteins were transferred from the gel to a polyvinylidene difluoride (PVDF) membrane by wet transfer at 100 V for 90 min (Bio-Rad Laboratories). PVDF membranes were rinsed in distilled water three times for 5 min each before blocking. Blocking was performed in 1× TBST (137 mM NaCl; 2.7 mM KCl; 19 mM Tris Base; 0.2% Tween 20) with 3% BSA for 4 h at room temperature. Primary anti-ATG16L1 antibody (1 ug/mL; TA306513, Origene, Rockville, MD) and secondary anti-rabbit IgG antibody (1:100,000 dilution; A0545, Sigma) were diluted in TBST with 1% BSA and 0.1% Tween 20 and incubated with the membrane overnight at 4°C and for 90 min at room temperature, respectively. Protein detection was performed with SuperSignal West Pico Kit Chemiluminescent Substrate (Thermo Fisher Scientific) using the ChemiDoc XRS+ System with Image Lab Software 1708265 (Bio-Rad).

Atg16l1 antibody was stripped from the membrane using Restore stripping buffer (Thermo Fisher Scientific) and the membranes were reprocessed for detection of β-actin protein. Briefly, membranes were washed in room temperature 1× TBS and incubated in stripping buffer for 15 min with agitation. Membranes were rinsed under cold tap water and washed in 1× TBST (0.1% Tween) three times for 10 min each before blocking in 1× TBST with 3% BSA for 4 h. Primary anti-β-actin antibody (1:2,500 dilution; ab8227; Abcam, Cambridge, UK) and secondary anti-rabbit antibody (1:100,000 dilution; A0545) were diluted in 1× TBST with 1% BSA for incubation at room temperature for 1 h and 90 min, respectively. Protein detection was performed as previously described.

### Intestinal Permeability

Four groups of rats (SD WT, F344 WT, em2 HET, and T300A HET) were evaluated for intestinal permeability. Forty-eight, 6-wk-old rats (6 males and 6 females for each group) were food and water fasted for 12 h for baseline intestinal permeability analysis. After fasting, rats were weighed and immediately gavaged with 50 mg/kg body weight fluorescein isothiocyanate (FITC) dextran (4,000 Da, 50 mg/mL in sterile water; Sigma). Four hours after gavage, animals were humanely euthanized by CO_2_ asphyxiation and cardiac exsanguination. Three milliliters of blood were collected into serum gel separator tubes (SST) with clot activator (Becton Dickinson, Franklin Lakes, NJ) and shielded from light. Tubes were inverted gently 6 times and placed upright at room temperature for 30 min to ensure proper clot formation. Serum was separated by centrifugation at 1,300 *g* for 12 min in a swinging-bucket rotor centrifuge. Serum (500 µL) was collected into a new microcentrifuge tube and diluted with equal parts sterile 1× PBS.

Diluted serum (100 µL) was added to a Greiner CELLSTAR black polystyrene plate with black polystyrene flat bottoms (Sigma) in triplicate. Concentration of FITC in individual serum samples was determined by fluorescence (excitation of 485 nm and emission of 528 nm) on the SpectraMax M3 microplate reader with SoftMax Pro 6.2 microplate data analysis software. Serially diluted FITC-dextran (0, 125, 250, 500, 1,000, 2,000, 4,000, and 8,000 ng/mL) in 1× PBS was used as standards. Standards were run in triplicate to ensure proper linear concentration.

### Histology

Ileum and proximal colon from six HET T300A rats and six WT littermates (3 males and 3 females per group) were collected for histologic analysis. Fresh tissues were fixed in 10% formalin for at least 24 h before being embedded in paraffin. Slides of each tissue were processed for analysis using a hematoxylin and eosin stain (H&E), alcian blue stain at pH 2.5 with a periodic acid-Schiff (PAS) counterstain, and lysozyme immunohistofluorescence (IFA). H&E and alcian blue/PAS measurements and cell counts were conducted on an Olympus BX41 microscope (Shinjuku, Tokyo, Japan) and images were captured using the Excelis HDS camera and monitor system (Accu-Scope, Commack, NY). IFA measurements were conducted on a Leica DMI4000 B automated inverted microscope and Leica TCS SPE confocal using the Leica Advanced Fluorescence application suite software (Wetzlar, Germany).

H&E and alcian blue/PAS staining was performed by IDEXX BioAnalytics histology services (Columbia, MO). Ileum and proximal colon were assessed with H&E stain to observe differences in villus length and crypt height. The length of each villus was measured from the top of the villus to the crypt transition (the invagination between two villi). Crypt height was measured from the crypt transition to the muscular layer. The lengths of 50 villi and heights of 50 crypts were measured by a blinded laboratory animal veterinarian trained in laboratory animal histology, in triplicate on three separate days (50 crypts measured randomly for three total counts, taking the average per day), per animal. Alcian blue and PAS stain was used for both ileal Paneth cell counts and ileal and proximal colon goblet cell counts.

Lysozyme IFA was used to observe differences in lysozyme staining within Paneth cells. Deparaffinization and rehydration of tissues was completed using the Novus Biologicals protocol for chromogenic immunohistochemistry staining of paraffin-embedded tissue. Rehydrated slides were rinsed in cold tap water and placed in antigen retrieval buffer (10 mM Tris Base, 1 mM EDTA solution, 0.05% Tween 20, pH 9.0) overnight in a 60°C water bath. Slides were cooled to room temperature and rinsed in 1× PBST (0.05% Tween 20) two times for 5 min each and blocked in 1× TBS with 10% normal goat serum (NGS) and 3% bovine serum albumin (BSA) for 4 h at room temperature. Slides were incubated in rabbit lysozyme polyclonal primary antibody (1:200 dilution; 15013-1-AP, Proteintech, Chicago, IL) and goat anti-rabbit IgG (H + L) Alexa Fluor Plus 488 secondary antibody (1 ug/mL; A32731, Invitrogen) diluted in 1% BSA blocking buffer overnight at 4°C and for 1 h at room temperature, respectively. Slides were mounted using VECTASHIELD HardSet Antifade Mounting Medium (Vector Laboratories, Burlingame, CA) and kept in the dark at 4°C for up to 1 wk before analysis.

### Microbiome

#### Fecal samples.

Twelve HET T300A rats and twelve WT littermates (equal numbers of each sex) were used for fecal microbiome analysis. Fecal samples were collected from each animal beginning at 4 wk of age and then 6 wk and 12 wk later. Individual rats were placed in empty autoclaved cages between the hours of 07:00 AM and 09:00 AM and allowed to defecate. Fecal pellets were collected with autoclaved, sterilized toothpicks, and placed into sterile microcentrifuge tubes. Samples were immediately frozen and stored at −80°C.

#### DNA extraction from feces.

Bacterial DNA was extracted according to a published protocol (21). Previously frozen fecal samples (30 mg) were placed in 800 μL of lysis buffer (500 mM NaCl, 50 mM tris-HCl, 50 mM EDTA, and 4% SDS), homogenized for 5 min at speed level 12 using the Bullet Blender Storm Tissue Homogenizer (Next Advance), and incubated at 70°C for 20 min. Samples underwent centrifugation at 5,000 *g* for 5 min at room temperature. The supernatant was mixed with 200 μL of 10 mM ammonium acetate, incubated on ice for 5 min, and centrifuged at 16,000 *g* for 10 min at room temperature. An aliquot of supernatant (750 μL) was mixed with equal volumes of chilled isopropanol and incubated for 30 min on ice. Samples were centrifuged at 14,000 *g* and 4°C for 15 min to pellet DNA. Pellets were rinsed twice with 70% ethanol and re-suspended in 150 μL Tris-EDTA. Proteinase K (15 μL) and buffer AL (200 μL; DNeasy Kit, QIAGEN) were added and samples were incubated at 70°C for 10 min. Then 100% ethanol (200 μL) was added and samples were transferred to a QIAGEN DNeasy spin column and processed per the manufacturer’s instructions for DNA purification (DNeasy Kit, QIAGEN). Samples were eluted in 100 μL EB buffer (QIAGEN) and dsDNA yield was measured using Qubit dsDNA BR assay kits and fluorometry (Qubit 2.0, Life Technologies, Carlsbad, CA).

#### Genomic library construction and 16S rRNA sequencing.

Library construction and sequencing was performed at the University of Missouri DNA Core facility. All DNA samples were normalized for PCR amplification. Bacterial 16S ribosomal DNA libraries were constructed by amplification of the V4 hypervariable region of the 16 s rRNA gene with universal primers (U515F/806R) flanked by Illumina standard adapter sequences and the following parameters: 98°C (3 min) + [98°C (15 s) + 50°C (30 s) + 72°C (30 s)] × 25 cycles +72°C (7 min). Amplicons were pooled for shotgun metagenome sequencing by Illumina MiSeq and V2 chemistry with 2 × 250 basepair paired end reads, as described previously (22).

#### Informatics.

Informatics analysis was conducted at the MU Informatics Research Core Facility (Columbia, MO). Quality-controlled reads were merged into contiguous sequences (contigs) using FLASh software (23). Qiime v1.8 software (24) was used to remove chimeric sequences and remaining contigs were binned and assigned to operational taxonomic units (OTUs). Taxonomic assignment was conducted using BLAST (25) against the SILVA database (26) of aligned microbial 16S rRNA gene sequences. Principle component analyses were performed on non-transformed OTU relative abundance data using Past v3.2 (27).

#### Statistical analysis.

All results were considered statistically significant for *P* values ≤0.05. Principal component analysis was performed using a nonlinear iterative partial least squares algorithm to evaluate β-diversity and its association with sex and genotype (WT or HET) through Metaboanalyst (28, 29). Statistical analysis for measures of intestinal permeability were performed by two-way ANOVA examining the effect of sex and genotype on intestinal permeability. Statistical analysis for histologic measurements were performed using the data analysis tools in Microsoft Excel 2016. Differences for histology were determined using two-tailed independent *t* tests for genotype (WT and HET).

## RESULTS

### Mutational Analysis of *Atg16l1* Genome Edited Rats

**Figure 1.**
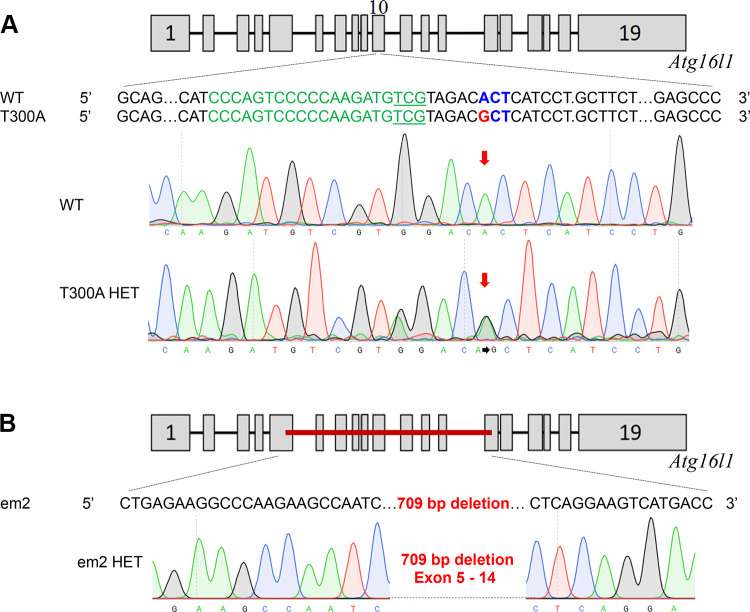
Mutational analysis of the genetic alterations in *Atg16l1* in the original em2 and T300A rat strains. The nucleotide sequence of each mutant *Atg16l1* allele was compared to the wild type *Atg16l1* sequence*. A*: the rat *Atg16l1* gene has 19 exons. The T300A allele has a single basepair change (A to G) in exon 10 (arrow). *B*: The em2 allele has a 709 bp deletion spanning exons 5 through 14.

**Figure 2.**
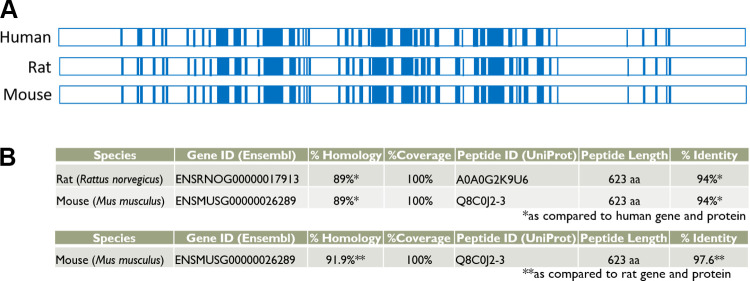
Comparison of human, rat, and mouse *Atg16l1*. *A*: comparison of genomic organization of the *Atg16l1* genes. Blue areas represent exons; white areas represent introns. Adapted schematics of *Atg16l1* for human (Uniprot; E7EVC7), rat (Uniprot; D3ZFK6), and mouse (Uniprot; G9M4M6). All 19 exons are represented in the human, mouse, and rat full-length transcripts. *B*: comparison of homology at the DNA level and identity at the protein level for rat and mouse compared to human (*top*) and mouse compared to rat (*bottom*).

The knockin modification in F344-*Atg16l1^em8Rrrc^* introduces the human CD susceptibility variant (T300A) into the rat *Atg16l1* gene. The single adenine to guanine substitution in exon 10 creates a nonsynonymous codon change that results in replacement of amino acid threonine with alanine but does not affect the coding length of the full-length transcript (Fig. 1). The knockout modification in SD-*Atg16l1^em2Rrrc^* results in a 709 basepair deletion starting in exon 5 and ending in exon 14. In the course of intercrossing heterozygous animals over the past 4 yr, we noted that neither strain produced rats that were homozygous for their respective genetic alterations, even though rats for both lines bred well and produced litter sizes within the expected averages for their respective genetic backgrounds (Table 1). Consequently, all studies have been carried out in heterozygous animals and their wild-type littermates. This apparent homozygous lethality has not been seen in humans or mice carrying the T300A mutation. (16) and was unexpected since the genomic and peptide sequences for *Atg16l1* are highly similar for humans, rats, and mice (Fig. 2).

**Table 1.** Genotypes for em2 and T300A litters from HET × HET matings

Rat Strain	Total No. of Pups	WT	HET	Hom	Litters
SD-*Atg16l1*^em2Rrrc^	221	85	136	0	29
F344-*Atg16l1*^em8Rrrc^	409	192	217	0	52

HET, heterozygous; HOM, homozygous; WT, wild type.

### Wild Type *Atg16l1* RNA Transcript Analysis

**Figure 3.**
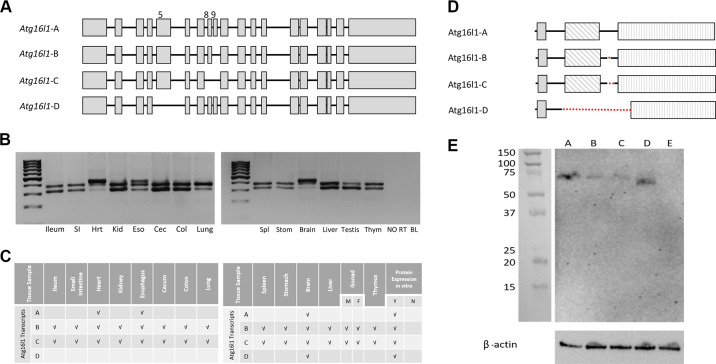
Wild type (WT) splice variant DNA and protein analysis. *A*: representative schematic of the four WT splice variants of rat Atg16l1. Gray boxes designate exons. *B*: representative agarose gel images depicting splice variants detected in different tissues. *C*: summary of WT splice variants detected in various tissues. *D*: representative schematic of predicted protein isoform for each splice variant. Dotted lines denote the area of each protein isoform missing relative to the full-length protein (Atg16l1-A). shaded box, ATG5 binding motif; diagonal hashmark box, coiled coil domain; vertical hashline box, WD40 repeat domains (7 total). *E*: representative Western blot image showing protein expression when DNA constructs coding for each splice variant were transfected into HEK293 cells. A, Atg16l1-A; B, Atg16l1-B; C, Atg16l1-C; D, Atg16l1-D; E, nontransfected HEK293 cells (negative control). Ladder: Precision Plus Protein Kaleidoscope Prestained Protein Standards (1610375; BioRad); Loading control: β-actin.

Four WT splice variants of *Atg16l1* were identified by RT-PCR using overlapping primers (Supplementary Table S2) and confirmed in both F344 and SD rats. From start to stop codon, these transcripts include: *Atg16l1*-A (full length, 1872 bp), and three alternative transcripts: *Atg16l1*-B (no exon 9; 1824 bp), *Atg16l1*-C (no exons 8 and 9; 1767 bp), and *Atg16l1*-D (no exon 5; 1620 bp) (Fig. 3*A*). *Atg16l1*-B and *Atg16l1*-C were present in all tissues tested. *Atg16l1*-A was only present in brain, heart and esophageal tissue and *Atg16l1*-D was only expressed in brain tissue (Fig. 3, *B* and *C*). Alternative transcripts *Atg16l1*-C and *Atg16l1*-D have not been reported previously in the rat.

### In Vitro Expression of Atg16l1 Protein Isoforms

Full length rat Atg16l1 protein contains 623 amino acids (*Atg16l1* -A; Fig. 3*D*). Individual splice variants code for protein isoforms of 607 (*Atg16l1*-B), 588 (*Atg16l1*-C) and 540 (*Atg16l1*-D) amino acids, respectively. It is not possible to effectively resolve these various isoforms by electrophoresis due to their similar sizes. To verify that protein could be produced from each splice variant, we cloned the coding region representing each alternative transcript into a mammalian expression vector and performed transfections in cell culture. All four *Atg16l1* transcripts were translated into protein in vitro in HEK293 cells (Fig. 3*E*).

### Intestinal Permeability of em2 and T300A Rats

**Figure 4.**
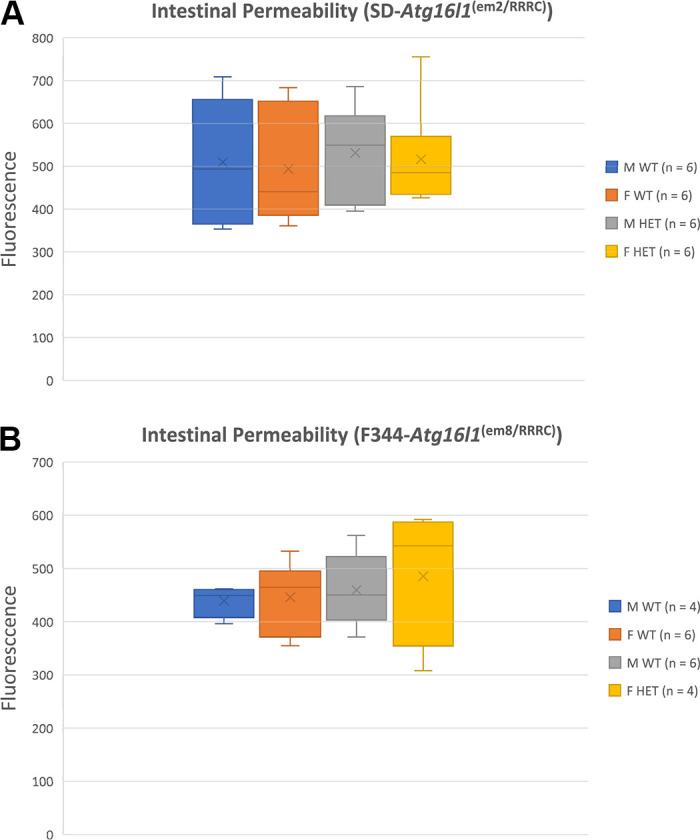
Fluorescein isothiocyanate (FITC) fluorescence for wild-type (WT) and heterozygous (HET) em2 (*A*) and T300A (*B*) rat strains. Average FITC fluorescence in relative fluorescence units (RFU) ± standard deviation. M, male; F, female; WT, wild type; HET, heterozygous; *n* = 6 for each group; samples were run in triplicate.

One of the most common theories on the initiation of IBD and CD is that damage to the epithelial lining of the gastrointestinal tract causes breaks in the luminal barrier and increases GI permeability thereby increasing the capacity for unwanted luminal bacteria to enter the gut tissues and cause inflammation (30). Therefore, we wanted to investigate whether the *Atg16l1* em2 and T300A mutations fit this narrative and cause increased permeability. The non-digestible fluorescein isothiocyanate (FITC dextran) molecule passively crosses the intestinal epithelium and, when measured in the plasma via fluorescence, represents a measure for paracellular permeability of the intestinal epithelium. We found that 6-wk-old HET rats from the em2 and T300A strains do not show increases in intestinal permeability over WT littermates (Fig. 4). There were no statistically significant differences for two-way ANOVA examining the effect of sex and genotype on intestinal permeability for either em2 (genotype *P* = 0.58; sex *P* = 0.78) or T300A (genotype *P* = 0.58; sex *P* = 0.78) rat strains. The retention of epithelial integrity as shown by normal intestinal permeability in both the HET em2 and HET T300A rats mimic findings in human patients with the T300A variant who also do not show an increased intestinal permeability (31).

### Intestinal Histology of T300A Rats

**Figure 5.**
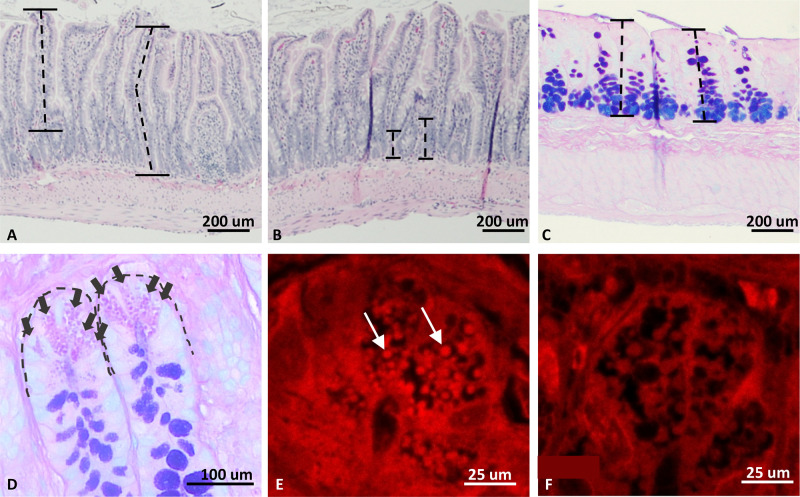
Representative histologic images from intestinal characterization of the T300A rat strain. *A*: villus length measurement (M WT ×100; H&E). Villi were measured parallel to the center of the villus from the luminal tip to the crypt transition. *B*: crypt height measurement (F WT ×100; H&E). Crypts were measured from the crypt transition to the muscular layer. *C*: colonic mucosal thickness (M WT ×100; Alcian blue/PAS). *D*: Paneth cell (PC) count (M WT; ×400; Alcian blue/PAS). *E* and *F*: lysozyme IFA of ileal crypt PC. F WT, ×630 (E) and F HET, ×630 (*F*) HET rats exhibit inherent defects in PC granule packaging and number of granules present within the cytoplasm. Dotted lines (*A–C*) represent examples of how measurements were taken. Arrows (*D*) highlight individual PCs. Arrows (*E*) mark a few of many granules present. M, males; F, female; WT, wild type; HET, heterozygous.

The gold standard for characterizing IBD in animal models is to visualize changes to the gastrointestinal tract at the macroscopic and microscopic level. However, to understand any changes that may occur by subjecting our T300A model to environmental triggers of IBD, we first need to understand the changes to the model due solely to the underlying genetic modification. It has been shown that samples of pre-disease human tissue display changes to Paneth cell morphology and granulation pattern, and we wanted to investigate whether this was the case for our rat model as well. Samples of the ileum and proximal colon were taken to evaluate changes to the intestinal epithelium, including ileal villus length, ileal crypt height, colonic mucosal thickness, and ileal Paneth cell counts and granulation patterns (Fig. 5). Six rats of each genotype (equal sexes) were evaluated, and the same cohort of animals was used for all histologic measurements.

**Figure 6.**
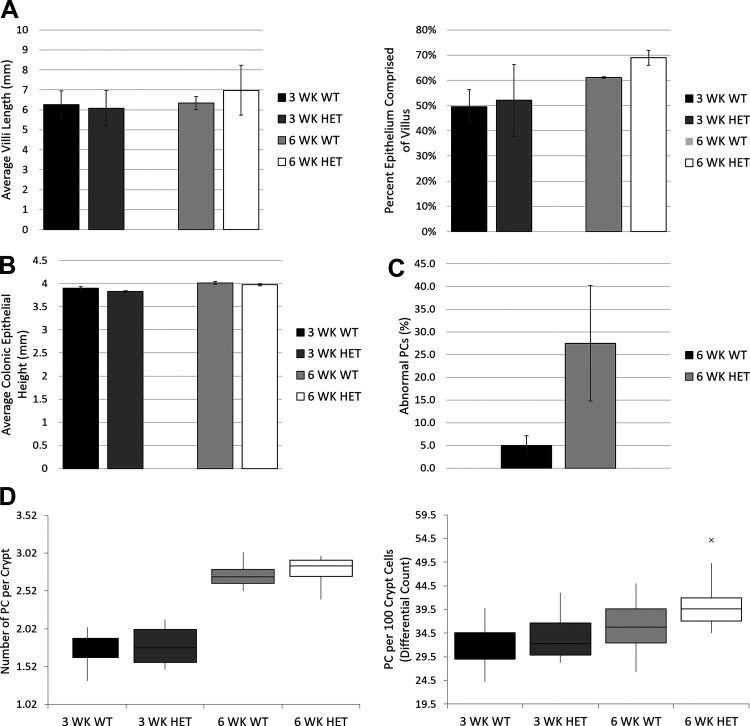
Quantitative intestinal histology in T300A strain. *A*: villus length as measured from the tip of the villus (luminal side) to the beginning of the crypt transition as measured by average villus length for WT and HET males and females and percent of epithelium comprised of the villus (villus length/villus length + crypt height). *B*: average colonic epithelium height as measured parallel from the luminal surface of the colonic mucosa to the base of the epithelium in contact with the muscular layer. *C*: abnormal Paneth cells (PC) on lysozyme IFA with results measured as abnormal (yes) or normal (no) and reported as the percentage of cells with abnormal PC granulation. *D*: PC counts as measured by both number of PC per crypt and a differential count of PC per 100 crypt cells. *n* = 6 for all groups; × in *D* represents a single outlier for the 6 WK HET group. Student’s *t* tests between 3 WK rats and between 6 WK rats used. WK, weeks.

Ileal villus length and crypt height as well as colonic mucosal thickness were analyzed using a H&E stain (Fig. 5, *A* and *B*). We found no statistically significant differences between WT and HET animals for either measurement at 3 wk (*t* = 2.31, *P* > 0.05) or 6 wk (*t* = 2.26, *P* > 0.05) of age (Fig. 6*A*). Fifty individual measurements were taken, in triplicate (50 randomly selected measurements per animal were taken 3 separate times on 3 separate days), for colonic mucosal thickness, measuring perpendicular from the luminal surface to the submucosal layer (Fig. 5*C*). Again, we found no significant differences in mucosal thickness between WT and HET rats at either 3 wk (*t* = 1.31, *P* > 0.05) or 6 wk (*t* = 1.19, *P* > 0.05) of age (Fig. 6*B*). These findings phenocopy those seen in healthy tissues from human patients with the T300A variant (16).

Paneth cell counts were performed using an alcian blue stain (pH 2.5) with a periodic acid-Schiff (PAS) counterstain (Fig. 5*D*). Twenty-five crypts per animal were evaluated, in triplicate, only if aligned along the longitudinal axis such that the lumen of the crypt could be seen along its length. Counts were performed at a constant magnification (×400) in two ways: *1*) by counting the number of Paneth cells per crypt; and *2*) by a differential count of at least 500 crypt cells with the results expressed as Paneth cells per 100 crypt cells. No significant differences were shown in the frequency of Paneth cells between WT and HET rats at 3 wk or 6 wk of age, regardless of the counting method used (per crypt at 3 wk, *t* = 1.69, *P* > 0.05; 6 weeks, *t* = 1.69, *P* > 0.05; or per 100 crypt cells at 3 wk, *t* = 1.70, *P* > 0.05; 6 wk, *t* = 1.70, *P* > 0.05; Fig. 6*D*).

Finally, we used lysozyme IFA to determine whether our 6-wk-old HET rats had greater expression of abnormal Paneth cells than WT littermates. Fifty Paneth cells per animal, in triplicate, were counted and abnormal granulation, defined as aberrant and disorganized granules, was recorded as either yes or no for each cell (Fig. 5, *E* and *F*). We noted a significant increase in the number of abnormal Paneth cells in tissues from HET rats (*t* = 20.69, *P* < 0.0001) (Fig. 6*C*). Instead of the distinct, bright, spherical granules located within the cytoplasm of most WT Paneth cells, many HET cells contained diffuse, finely granular staining with no visible granules present within the cytoplasm of the cell. This abnormal Paneth cell finding is consistent with results from human patients with the T300A CD susceptibility variant as well as mouse models with a hypomorphic variant of *Atg16l1* (10, 16).

### Microbiome Analysis of T300A Rats

**Figure 7.**
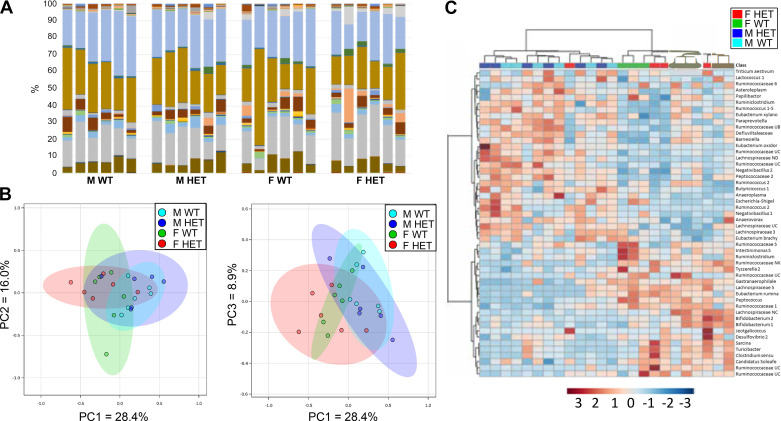
Gut microbiome analysis for 16-wk-old WT and HET T300A rats. *A*: stacked bar charts of genus-level OTUs for each rat per group. Each color represents a unique OTU in each group. *B*: principal component analysis plots. The amount of distance between 95% confidence ellipses show the amount of difference between groups. No significant differences were found. *C*: cluster heat map grouping samples based on the 25 most common OTUs present in the dataset from rats of WT and HET genotypes (*n *=* *6/group, *upper right*) with samples arranged according to an unweighted pair group method with arithmetic mean (UPGMA) algorithm. HET, heterozygous; WT, wild type.

Previous studies have shown that IBD risk alleles can affect gut microbial composition (32). Due to the role of *Atg16l1* in autophagy and the processing of intracellular bacteria, we evaluated whether the T300A variant could alter the gut microbiota in 16-wk old conventionally housed HET T300A rats versus their WT littermates. This age point was chosen because it falls well within the age range of “adult” for F344 rats, and CD tends to be a disease developed in late adolescence to young adulthood. We found no apparent significant differences between the genus-level OTU (operational taxonomic unit; clusters of organisms grouped by DNA sequence similarity which represent pragmatic proxies for “genus”) for WT and HET T300A rats (Fig. 7*A*). Principal component analyses in the PC1 × PC2 and PC1 × PC3 directions also reveal overlapping, and therefore similar, microbiome compositions for each group (Fig. 7*B*). One-way PERMANOVA for the effect of sex and genotype on microbiome revealed no statistically significant difference between WT and HET groups for males or females. On heat map analysis, which compares the 25 most prevalent OTUs in our study between individual rats from all experimental groups, we found that our males and females grouped together based on sex, regardless of genotype. This suggests a difference in gut microbiota composition between males and females in our study (Fig. 7*C*). This microbiome difference between sexes has been confirmed in past studies and is not unique to our model; therefore, further qualitative and quantitative analysis of males versus females was not conducted (22, 33, 34).

## DISCUSSION

### Wild Type *Atg16l1* in the Rat

We describe one of the first characterizations of wild-type *Atg16l1* in the rat. The full-length wild type rat *Atg16l1* gene contains 19 exons, the same number of exons found in the human and mouse full-length genes. We identified four wild-type rat *Atg16l1* alternative transcripts, all of which contain the codon involved in the T300A susceptibility variant. Two of these transcripts, *Atg16l1*-C (missing exons 8 and 9) and *Atg16l1*-D (missing exon 5) have not been previously described. The human *ATG16L1* gene has nine protein-coding alternative transcripts, four of which are the same as those we have found in the rat: one full-length transcript, one missing exon 9, one missing exons 8 and 9, and one missing exon 5. The mouse also has four protein-coding alternative transcripts, three of which are the same as those found in the rat: one full-length transcript, one missing exon 9, and one missing exon 5. Interestingly, the T300A CD susceptibility variant falls within exon 9 of the human and mouse genes but just at the beginning of exon 10 in the rat. This means that, although all wild-type rat *Atg16l1* alternative transcripts carry the susceptibility variant, some wild type alternative transcripts of human and mouse do not. These transcripts which are not affected by the T300A variant in humans and mice may compensate for those that are and may provide an answer as to why humans and mice can be homozygous viable for the variant (16) although it is embryonic lethal in the rat.

By extracting total RNA from healthy rat tissue samples from different organs, we showed that the four wild type alternative transcripts are differentially expressed in different tissues, with heart and brain being the only tissues to express all four transcripts. We found that all four of the wild-type rat *Atg16l1* alternative transcripts could produce protein in vitro when individually transfected into HEK293 cells in a plasmid with an overexpression promotor (CMV). As all four transcripts code for a protein product, it suggests that there are four protein isoforms with the potential to function at some capacity during autophagy. Future studies will be aimed at evaluating the function of each isoform during the autophagy process to understand why we see differential tissue expression of the various alternative transcripts.

### Knockout *Atg16l1*

We have generated a new knockout rat strain with a 709 bp deletion in *Atg16l1* (SD-*Atg16l1^em2Rrrc^*). This is the first rat strain described to date with a deletion in the *Atg16l1* gene. Based on the location of the deletion, we predict that the protein produced by this mutant allele will have a disruption beginning in the terminal portion of the coiled coil domain that causes the downstream β-transducin (WD40) repeats of the Atg16l1 protein to be missing, leading to a null allele. We were able to produce 29 litters from HET × HET matings of the em2 strain, none of which produced a homozygous pup (Table 1), suggesting that this genotype is embryonic lethal and that *Atg16l1* plays a vital role during embryonic development. Interestingly, although not embryonic lethal, homozygous mutant mice that carry a mutation that results in a truncated version of the Atg16l1 protein lacking the entire coiled coil domain die within 1 day of birth (35). To date, there are no reports of human *ATG16L1* null alleles implicated in CD susceptibility. Although we chose not to further characterize this particular mutant rat stock beyond assessing gut permeability, it carries a unique allele not found in other *Atg16l1* rodent models and it has utility for studies to investigate the biological role of Atg16l1 and how the autophagy process is impacted in the absence of intact Atg16l1.

### *Atg16l1* T300A Variant

We have generated and characterized a rat model carrying the T300A susceptibility variant for CD in the *Atg16l1* gene. This is the first rat strain described to date that carries an IBD susceptibility variant in the *Atg16l1* gene. We maintained a breeding colony of HET × HET T300A rats, resulting in 52 litters of pups. No pups born from these crosses were homozygous for the T300A variant, suggesting that this single amino acid substitution is embryonic lethal. Interestingly, the number of HET animals produced in T300A HET × HET matings was also less than expected. It is possible that inheritance of the T300A allele is associated with embryonic lethality in both homozygous and heterozygous rats. Alternatively, there may be some selective disadvantage in gametes that carry the T300A allele that results in no homozygous and fewer heterozygous offspring being produced. Further studies would be necessary to determine the reason for the skewed genotypic ratios observed in these crosses. Nonetheless, this strain breeds well which allows sufficient numbers of animals to be generated for studies. The embryonic lethality seen in T300A rats is not seen in humans or mice carrying this same genetic alteration and it represents one of the most interesting findings of our studies. As discussed earlier, this difference in lethality may be due to differences in the *Atg16l1* splice variants seen among the three species and presents an opportunity to explore this biological phenomenon to potentially gain valuable insight that could have beneficial implications for CD research.

An allelic series is a collection of mutations in a gene that alter different parts of the gene sequence, and these can be used to dissect the impact of individual regulatory components of genes as well as functional domains within proteins. The various mutations in human, mouse and now rat provide a powerful allelic series in which to investigate the impact of alterations in the different regions of the *Atg16l1* gene, splice variants, and protein. The rat is the only known species to date that is homozygous lethal for the single nonsynonymous *Atg16l1* T300A polymorphism. Therefore, we believe our findings of embryonic lethality indicate a fundamental biological difference among human, mouse and rat that may have important implications for understanding the basic functions of *Atg16l1.*

Crohn’s Disease is multifactorial, genetic variants represent risk factors only and it is the combined genetic and environmental liability that push the individual over the threshold that leads to disease. Although it will not be possible to study T300A homozygosity in the rat, some human patients are heterozygous, not homozygous, for this SNP (36) and therefore homozygous lethality does not lessen the relevance of the rat allele for Crohn’s disease research. In addition, by mating the T300A strain with other rat strains carrying different genetic modifications, we have the ability to combine the T300A risk allele with other known risk alleles in rats to model human genetics related to disease.

Phenotypic characterization of T300A rats focused on whether this model presents with alterations in gut permeability, microbiota composition, or histologic features—three of the most common metrics in IBD and CD studies. It is important to understand any phenotypic characteristics that present solely due to inheritance of the susceptibility variant itself in the absence of environmental triggers of disease.

Although intestinal permeability is a common inciting cause of inflammatory bowel disease (37), human patients carrying the *Atg16l1* T300A susceptibility variant do not have inherent increases in intestinal permeability (31). Likewise, our rat strains do not have inherent increases in intestinal permeability as measured by the FITC-dextran permeability assay. The mechanism by which *Atg16l1* is thought to initiate CD lies within its role in regulation of homeostasis of the gastrointestinal lumen, not permeability (38). *Atg16l1* is vital for proper exocytosis of Paneth cell (PC) granules which encapsulate α-defensins, lysozyme, and other antibacterial modulators and release them into the intestinal lumen as part of the mucosal immune barrier. When the T300A variant is present, ATG16L1 protein is sensitive to caspase-3 mediated cleavage. Increased cleavage results in reduced concentrations of ATG16L1 protein, limiting the release of antibacterial modulators into the intestinal lumen. The luminal microbiome, now unburdened by immunomodulators, has a high propensity for dysbiosis, which is hypothesized to be the cause of CD in susceptible individuals. Thus, the finding of no permeability changes is unsurprising.

Analysis of the microbiome of T300A WT and HET rats shows differences between males and females; however, this sex-dependent difference is not unique to our T300A strain and is a common feature of many rat models (39). There does not appear to be any significant differences in the microbiome of 16-wk-old WT and HET rats carrying the T300A variant. It is surprising that we did not see a gut microbiota shift related to genotype in our rat model when differences exist in predisease patients with CD with the *Atg16l1* susceptibility variant; however; many disparities between human and rat host factors exist, such as diet, environment, and features of the gastrointestinal system itself (differences in small intestine: colon ratios, size of the cecum, etc.) which could explain why the rat gut microbiota may be less susceptible to changes based on the T300A variant alone. There are also several differences between the rat and human gut microbiota that could explain its heartiness against T300A variant changes (40). Outbred rats have a higher prevalence of *Prevotella* and fecal lactate than humans. The gut microbiota of rats appears to be dominated by *Bacteroidetes* over *Firmicutes*, whereas, human gut microbiota composition demonstrates equal to slightly higher numbers of *Firmicutes*. Recently, it was found that gnotobiotically raised mice expressing the T300A variant that received feces from patients with active CD exhibit an increase in *Bacteroides* abundance compared to WT mice (41). This technology could be used in rat models as well to understand the implications of *Atg16l1* variants on the gut microbiota. Future studies in the T300A rat strain will seek, in part, to determine what microbiome changes are revealed in rats with active CD and whether they are comparable to human patients with active CD.

Intestinal histology from F344-*Atg16l1^em8Rrrc^* WT and HET rats shows PC abnormalities such as those seen in human patients and mice carrying the *Atg16l1* T300A variant (16). These PCs display diffuse lysozyme staining throughout the cytoplasm of the cells as shown by lysozyme IFA staining. These cells also display a significant decrease in granules (vesicles) within the cytoplasm, suggesting that production of α defensins and other host defense proteins packaged within granules in the PCs is working, but the packaging process is not. PCs function by exocytosis of their granules into the intestinal lumen to release these defense proteins to modulate the luminal environment (42). This process requires the membrane of the vesicle to fuse with the PC membrane. Without granule formation, these enzymes cannot escape the PCs and cannot perform their intended function. Further studies on the role of *Atg16l1* in PC packaging and exocytosis and the effect during CD are needed.

Overall, the *Atg16l1* T300A rat strain provides a valuable new animal model. Although we recognize that it will not be possible to study T300A homozygosity in the rat, it is still highly relevant as some human patients are heterozygous, not homozygous, for this single nucleotide polymorphism (SNP) (36). Although a mouse model carrying the *Atg16l1* T300A susceptibility variant exists, there are many benefits to a larger rodent model with characteristics mimicking human patients with CD. The larger size of the rat makes them preferable for applications that may involve tissue sampling or serial sampling. Rats are also a much easier model in which to perform surgery, and, given the high likelihood of patients with CD to undergo at least one surgical procedure to address disease symptoms (most commonly intestinal resection and anastomosis), this makes the rat an appealing CD model for future therapies and treatments. Like human patients with CD carrying the T300A variant, the T300A rat strain exhibits inherent abnormalities in PCs, including abnormal packaging of antibacterial lysozyme and an overall decrease in PC granules. Future studies using this model will examine the potential to induce CD-like lesions using known environmental triggers of CD. Overall, these new rat models will facilitate further studies into the mechanism of *Atg16l1* in autophagy and CD.

## SUPPLEMENTAL DATA

Supplemental material available at: https://doi.org/10.6084/m9.figshare.12949244.v1; https://doi.org/10.6084/m9.figshare.12949238.v1; https://doi.org/10.6084/m9.figshare.12949241.v1; https://doi.org/10.6084/m9.figshare.12949235.v1.

## GRANTS

This project was supported by funding from the National Institutes of Health, Office of the Director under Grants P40 OD011062, P40 OD011062-14S1, and T32 OD011126.

## DISCLAIMERS

The content is solely the responsibility of the authors and does not necessarily represent the official views of the National Institutes of Health.

## DISCLOSURES

No conflicts of interest, financial or otherwise, are declared by the authors.

## AUTHOR CONTRIBUTIONS

K.L.C., H.M., and E.C.B. conceived and designed research; K.L.C., H.M., and M.A.H. performed experiments; K.L.C., H.M., and M.A.H. analyzed data; K.L.C. interpreted results of experiments; K.L.C. prepared figures; K.L.C. drafted manuscript; K.L.C. and E.C.B. edited and revised manuscript; K.L.C., H.M., M.A.H., and E.C.B. approved final version of manuscript.
